# Predictor of Syncopal Recurrence in Children With Vasovagal Syncope Treated With Metoprolol

**DOI:** 10.3389/fped.2022.870939

**Published:** 2022-04-08

**Authors:** Chunyan Tao, Bowen Xu, Ying Liao, Xueying Li, Hongfang Jin, Junbao Du

**Affiliations:** ^1^Department of Pediatrics, Peking University First Hospital, Beijing, China; ^2^Department of Genetics and Endocrinology, Guangzhou Women and Children’s Medical Center, Guangzhou Medical University, Guangzhou, China; ^3^Department of Statistics, Peking University First Hospital, Beijing, China; ^4^Key Laboratory of Molecular Cardiovascular Sciences, Ministry of China, Beijing, China

**Keywords:** vasovagal syncope, children, syncopal recurrence, metoprolol, predictor

## Abstract

**Objective:**

To explore the predictors for syncopal recurrence in a pediatric population with vasovagal syncope (VVS) treated with metoprolol.

**Study Design:**

This study was conducted retrospectively among children suffering from VVS with or without syncopal recurrence. Data on the detailed medical history and auxiliary examinations were obtained from the electronic medical records. The risk factors for syncopal recurrence were studied by cox regression analyses and the corresponding best cutoff values were determined using receiver operating characteristic analysis. Kaplan–Meier curves were plotted to determine the trends of the syncopal recurrence-free survival rate.

**Results:**

Forty-two consecutive VVS children were enrolled in the study. At the end of a median follow-up duration of 9.0 (4.8, 19.1) months, 12 patients (29%) experienced ≥1 syncopal episode. Cox regression analyses revealed that the number of previous syncopal episodes before treatment was a risk factor for syncopal recurrence (hazard ratio = 1.027, 95% confidence interval 1.009 – 1.045, *P* = 0.003). Moreover, 4 previous syncopal episodes were certified as the best cutoff value, and the Kaplan–Meier curves showed that the syncopal recurrence-free survival rate over time in patients with > 4 previous syncopal episodes was significantly lower than that in patients with ≤4 episodes (*P* = 0.019 at the log-rank test).

**Conclusion:**

In a pediatric population with VVS while on the treatment of metoprolol, the number of previous syncopal episodes before treatment played a significant role in predicting syncopal recurrence.

## Introduction

Vasovagal syncope (VVS) is the most common subtype of syncope in the young. Furthermore, it is speculated that VVS constitutes more than 50% causes of syncopal events (1). Up to 15% of children are reported to experience at least one syncopal event before the end of adolescence, and the syncope recurrence rate is approximately 25–35% for a one-year period and 33–51% for a five-year period (2, 3). VVS is believed to be benign; however, recurrent episodes notably decrease the quality of life and increase the risk of severe injuries (4–6). Metoprolol, a β-adrenoceptor blocker, is considered a reasonable therapy on the basis of the commonly recognized mechanism of excessive sympathetic activity (7, 8). However, based on our experience, treatment with metoprolol could not ensure no recurrence of syncope in a substantial number of VVS cases (9). Indeed, syncopal attack greatly restricts daily life activities such as school and physical activities in those patients. Therefore, identifying the predictors of the recurrence of syncope is necessary for the clinical management and prevention. Thus, this research was designed to identify the predictors of syncopal recurrence in VVS of children with or without syncopal recurrence while on the metoprolol treatment.

## Study Subjects and Methods

### Study Subjects

This study authorized by the Ethics Committee of Peking University First Hospital was performed retrospectively. Patients younger than 18 years accompanied by their parents or other relatives visited the Pediatric Syncope Unit, Department of Pediatrics, Peking University First Hospital, China, from November 2011 to July 2019. For further diagnosis, they were assigned to be hospitalized. They were finally diagnosed with VVS with the exclusion of cardiogenic, neurologic or metabolic causes and psychogenic pseudo-syncope. The exact diagnostic criteria for VVS in childhood were previously described (10, 11). The detailed histories (medical history, family history and allergic history) and the results of auxiliary examinations [i.e., QT dispersion (QTd) and QTc dispersion (QTcd)] from an electrocardiogram and head-up tilt test (HUTT) results were obtained from the electronic medical records. We also analyzed the hemodynamic response data during the HUTT.

### Head-Up Tilt Test

Patients who refrained from any medication influencing autonomic nervous function for ≥5 half-life times and fasted for ≥4 h were assigned to complete the HUTT in the morning. The testing room was warm, dimly lit and quiet, and the necessary resuscitation equipment was available with the informed consent from the legal guardian/next of kin of the subjects. After emptying their bladders, the subjects were asked to lie quietly for 10–20 min on a tilt table (SHUT-100A, Standard, and ST-711, Juchi, China) which was controlled electronically. Then, they were passively tilted 60° for 45 min at most or the tilting process was discontinued once a positive response happened. The exact criteria for a positive response were that a subject was distressed by orthostatic intolerance symptoms like blurred vision, dizziness, sweating, headache or even syncope, accompanied by one of the following hemodynamic changes (12): (1) a significant hypotension, namely systolic blood pressure (BP) ≤80 mmHg, diastolic BP ≤50 mmHg or ≥25% decrease in mean BP; (2) an obvious bradycardia, namely heart rate (HR) <75 bpm, 65 bpm, and 60 bpm, respectively, in children at the age of 4–6 years, 6–8 years, and >8 years; (3) second or third-degree atrioventricular block and asystole for longer than 3 s; and (4) sinus arrest (12). On the basis of hemodynamic alterations during a positive response, the types of VVS were categorized as a vasodepressive pattern (an obvious decline in BP without notable reduction in HR), a cardioinhibitory pattern (a notable reduction in HR without obvious decline in BP), or a mixed pattern (a notable decrease in both BP and HR) (12). No medicine (e.g., isoproterenol or nitroglycerin) was administered during the test.

### QT Dispersion Measurement

QT dispersion, an index from electrocardiogram reflecting autonomic function, was defined as the maximum QT interval minus the minimum one and QTcd was defined as QTd corrected by HR using Bazett’s equation (QTcd = QTd/$\sqrt{R-R}$, in which R-R means the RR interval in seconds) (13). Naturally, the QT interval meant the interval between the QRS complex onset and the end point of the T wave (14–16). The QTd and QTcd data were obtained from standard 12-lead resting electrocardiograms, which were recorded at 25 millimeters/second. The intervals of QT and RR were measured manually by one investigator using a caliper, and they were determined as the mean values of three consecutive beats in each lead. If the end point of the T wave was not identified reliably, the lead would be eliminated from the subsequent analysis.

### Treatment and Follow-Up

All enrolled patients received the regimen of metoprolol [0.5 mg/kg/d, for 3.0 (2.0, 4.0) months] and health education empirically once the diagnosis of VVS was established. From the ending of treatment, they were followed up for a median duration of 9.0 (4.8, 19.1) months by telephone or outpatient visits. The follow-up was performed by a professionally trained investigator and the recurrence of syncope or presyncope was recorded in the electronic medical records. The first recurrence of syncope was the endpoint of this study. Patients with recurrent syncopal episode(s) during follow-up were recorded as “patients with recurrence,” otherwise recorded as “patients without recurrence.” All the tasks were conducted by one professionally trained researcher.

### Statistical Analyses

SPSS, version 21.0 (IBM, Armonk, New York, United States), was applied for data analyses. The normality of continuous variables was examined with the Shapiro–Wilk test. The data with normal distribution are listed as the mean ± standard deviation, otherwise as the median (interquartile range) and they were compared using the Student’s *t*-test or the Mann–Whitney *U*-test appropriately. Categorical variables are presented as numbers (percentages) and were compared using the chi-square test or the continuity correction. The data of patients with and without recurrent syncope were compared as described above. Univariate and multivariate Cox proportional hazards models were performed to assess the unadjusted and adjusted hazard ratios with 95% confidence intervals for some suspicious risk variables. The best cutoff values of continuous risk variables for syncopal recurrence were calculated by the receiver operating characteristic analysis. Kaplan–Meier curves for the cumulative rate free from recurrence were utilized to study the trends of patients suffering recurrent events over time, and the log-rank test was applied to assess the trends correspondingly. Differences with two-tailed *P*-value <0.05 were supposed to be statistically significant.

## Results

### Recurrent Syncopal Events During Follow-Up

**FIGURE 1:**
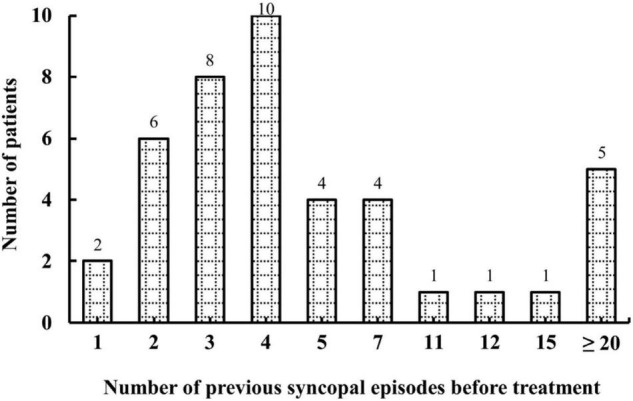
Number of patients with different syncopal episodes before treatment. The *y*-axis represents the number of patients; the *x*-axis represents the number of previous syncopal episodes before treatment. The median frequency was 4.0 episodes/person/year.

**FIGURE 2:**
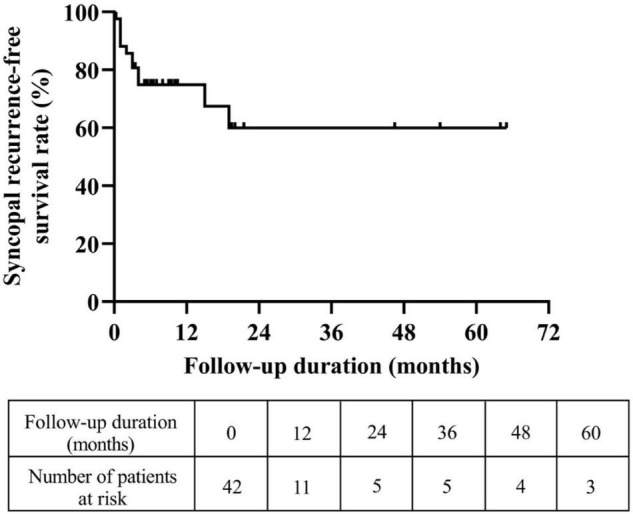
Trend of the syncopal recurrence-free survival rate in all eligible patients over time. The *y*-axis represents the survival rate for non-recurrence of syncope; the *x*-axis represents follow-up duration.

A total of 46 pediatric patients with VVS while on the metoprolol treatment were initially recruited in this study. However, 4 of them were lost to follow-up. At admission, 687 syncopal episodes in total were reported in a median symptomatic duration of 14.5 (4.0, 39.0) months. The median frequency was 4.0 (1.3, 21.0) episodes/person/year (Figure 1). At the end of a median follow-up duration of 9.0 (4.8, 19.1) months, 12 (29%) of the 42 patients experienced at least one recurrent syncopal event, and the median interval from the first recurrent syncope was 2.5 (1.0, 4.0) months. The Kaplan–Meier curve in Figure 2 presents the overall trend of syncopal recurrence in all included patients over time.

### Baseline Characteristics Between Patients With and Without Syncopal Recurrence

The baseline characteristics between patients with and without syncopal recurrence are listed in Table 1, and they were compared. Compared to patients without syncopal recurrence, those with recurrence had more previous syncopal episodes before treatment (*P* = 0.013) and higher supine mean BP (*P* = 0.016). No differences were found for the sex ratio, age at the first syncopal episode, age at the first visit of our department, duration of symptoms, triggers of syncope, prodromes, duration of unconsciousness, injury at syncope, history of allergy, family history of syncope, body mass index, other hemodynamics during the HUTT and the time to positive response, types of VVS, QTd, QTcd, treatment duration and follow-up duration (*P* > 0.05).

**TABLE 1:** The baseline characteristics of the study population and comparisons of patients with and without syncopal recurrence.

Parameters	All patients	Patients with syncopal recurrence		t/Z/χ ^2^value	*P*-value
		
		Yes	No		
Number [n (%)]	42 (100)	12 (29)	30 (71)	–	–
Female [n (%)]	29 (69)	8 (67)	21 (70)	0.000	1.000
Age at first syncopal episode (years)	9.6 ± 3.5	11.5 (10.3, 12.0)	9.2 ± 3.5	−1.359	0.174
Age at first visit of our department (years)	11.6 ± 2.5	12.6 ± 2.9	11.3 ± 2.3	−1.565	0.126
Duration of symptoms before treatment (months)	14.5 (4.0, 39.0)	22.4 ± 19.5	14.5 (3.0, 39.0)	−0.391	0.696
Triggers of syncope [yes, n (%)]	31 (74)	8 (67)	23 (77)	0.077	0.781
Prodromes [yes, n (%)]	39 (93)	12 (100)	27 (90)	0.224	0.636
Duration of unconsciousness [<1 min, n (%)]	13 (31)	4 (33)	9 (30)	0.000	1.000
Injury at syncope [yes, n (%)]	6 (14)	3 (25)	3 (10)	0.588	0.443
Number of previous syncopal episodes (times)	4 (3, 7)	6 (3, 44)	4 (2, 5)	−2.467	0.013
History of allergy [yes, n (%)]	13 (31)	2 (17)	11 (37)	0.805	0.370
Family history of syncope [yes, n (%)]	7 (17)	1 (8)	6 (20)	0.210	0.647
Body mass index (kg/m^2^)	18.7 (16.9, 23.3)	19.6 (17.7, 25.7)	19.7 ± 4.3	−1.114	0.265
Supine heart rate during head-up tilt test (beats/minute)	80 ± 12	79 ± 13	80 ± 12	0.218	0.829
Supine mean blood pressure during head-up tilt test (mmHg)	80 ± 8	85 ± 9	78 ± 7	−2.506	0.016
Positive heart rate during head-up tilt test (beats/minute)	100 ± 33	105 ± 33	98 ± 33	−0.670	0.507
Positive mean blood pressure during head-up tilt test (mmHg)	54 ± 9	51 ± 8	55 ± 9	1.426	0.162
Time to positive response during head-up tilt test (minutes)	27 (11, 34)	26 ± 13	23 (10, 36)	−0.405	0.686
Types of vasovagal syncope [vasodepressive type, n (%)]	33 (79)	10 (83)	23 (77)	0.004	0.953
QT dispersion (milliseconds)	25 (20, 31)	25 (20, 36)	25 (20, 30)	−0.183	0.855
QTc dispersion (milliseconds)	29 (22, 39)	29 ± 9	29 (22, 34)	−0.223	0.824
Treatment duration (months)	3.0 (2.0, 4.0)	3.5 ± 1.7	3.0 (2.0, 3.6)	−1.011	0.312
Follow-up duration (months)	9.0 (4.8, 19.1)	15.0 (4.3, 19.0)	8.0 (4.8, 19.1)	−0.599	0.549

### Predictors of Syncopal Recurrence in Patients With Vasovagal Syncope

Table 2 shows the possible associated factors for the recurrence of syncope. In univariate Cox regression analysis, age at the first visit of our department (hazard ratio = 1.313, 95% confidence interval 1.013–1.700, *P* = 0.039), the number of previous syncopal episodes before treatment (hazard ratio = 1.027, 95% confidence interval 1.009–1.045, *P* = 0.003) and supine mean BP (hazard ratio = 1.096, 95% confidence interval 1.022–1.177, *P* = 0.011) were recognized as possible associated factors for syncopal recurrence. Furthermore, the number of previous syncopal episodes before treatment was identified as an independent risk factor (hazard ratio = 1.027, 95% confidence interval 1.009–1.045, *P* = 0.003) when taken into the multivariate analysis, but other parameters that were included showed no predictive value for recurrence.

**TABLE 2:** Risk factors for syncopal recurrence from Cox regression analyses.

Parameters	Univariate analysis		Multivariate analysis	
		
	Hazard ratio (95% confidence interval)	*P*-value	Hazard ratio (95% confidence interval)	*P*-value
Female gender	0.955 (0.286–3.191)	0.940	–	–
Age at first syncopal episode (years)	1.163 (0.970–1.393)	0.103	–	–
Age at first visit of our department (years)	1.313 (1.013–1.700)	0.039	–	–
Duration of symptoms before treatment (months)	0.992 (0.972–1.013)	0.448	–	–
Triggers of syncope (yes)	0.537 (0.160–1.809)	0.316	–	–
Prodromes (yes)	24.861 (0.007–87551.961)	0.441	–	–
Duration of unconsciousness (<1 min)	1.162 (0.348–3.877)	0.808	–	–
Injury at syncope (yes)	1.972 (0.532–7.310)	0.310	–	–
Number of previous syncopal episodes (times)	1.027 (1.009–1.045)	0.003	1.027 (1.009–1.045)	0.003
History of allergy (yes)	0.338 (0.073–1.565)	0.165	–	–
Family history of syncope (yes)	0.508 (0.064–4.014)	0.521	–	–
Body mass index (kg/m^2^)	1.079 (0.983–1.183)	0.108	–	–
Supine heart rate during head-up tilt test (beats/minute)	0.992 (0.943–1.045)	0.770	–	–
Supine mean blood pressure during head-up tilt test (mmHg)	1.096 (1.022–1.177)	0.011	–	–
Heart rate at positive response of head-up tilt test (beats/minute)	1.007 (0.989–1.026)	0.433	–	–
Mean blood pressure at positive response of head-up tilt test (mmHg)	0.963 (0.905–1.024)	0.229	–	–
Time to positive response during head-up tilt test (minutes)	1.032 (0.984–1.082)	0.196	–	–
Types of vasovagal syncope (vasodepressive type)	1.607 (0.351–7.366)	0.541	–	–
QT dispersion (milliseconds)	1.008 (0.953–1.066)	0.789	–	–
QTc dispersion (milliseconds)	1.001 (0.951–1.054)	0.959	–	–
Treatment duration (months)	1.156 (0.836–1.598)	0.382	–	–

**FIGURE 3:**
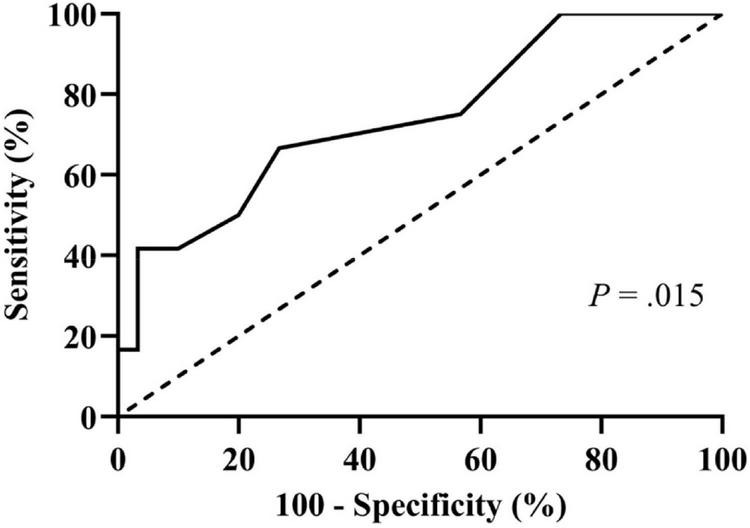
Receiver operating characteristic curve for determining the best cutoff for the number of previous syncopal episodes in predicting syncopal recurrence. The *y*-axis represents the sensitivity to predict the recurrence of syncope; the *x*-axis represents the false-positive rate (100%–specificity%). The 45 reference line of the chart indicates that the sensitivity and the false-positive rate are equal. The area under the curve was 0.743 with a 95% confidence interval 0.574 to 0.912 (*P* = 0.015).

**FIGURE 4:**
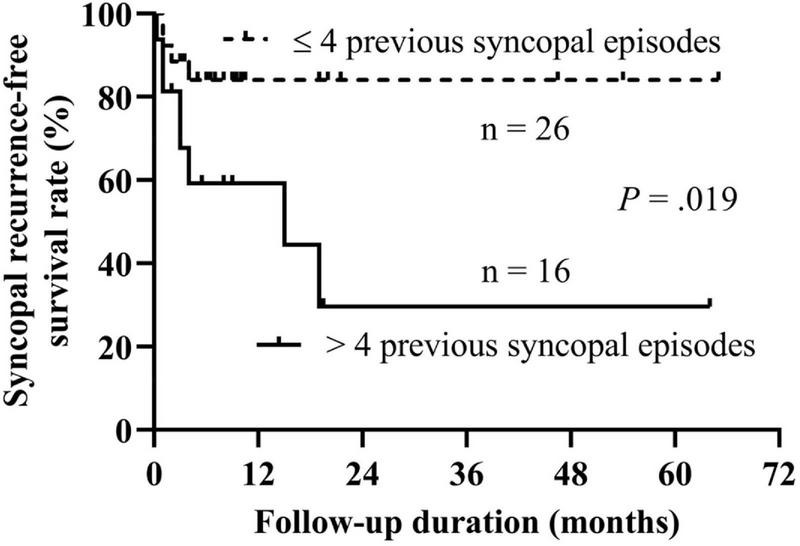
Kaplan–Meier curve analysis of the syncopal recurrence-free survival rate between patients with >4 previous syncopal episodes and with ≤4 previous sycopal episodes. The *y*-axis represents the survival rate for non-recurrence of syncope; the *x*-axis represents follow-up duration. The recurrence-free survival rate of syncope in patients with >4 previous syncopal episodes before treatment was significantly lower than that of patients with ≤4 episodes (*P* = 0.019 at the log-rank test).

Then, we performed the receiver operating characteristic curve analysis to decide the best cutoff number of previous syncopal episodes before treatment for predicting syncopal recurrence (Figure 3). The area under the curve was 0.743 (95% confidence interval 0.574–0.912, *P* = 0.015), and 4 previous syncopal episodes before treatment were determined as the best cutoff value for reaching the highest Youden index. Moreover, the Kaplan–Meier curves showed that the syncopal recurrence-free survival rate over time in patients with >4 previous syncopal episodes before treatment was much lower than that of patients with ≤4 episodes (*P* = 0.019 at the log-rank test, Figure 4).

## Discussion

To our best knowledge, this was the first study on the prediction of syncopal recurrence in children with VVS while on the treatment of metoprolol. We found that in the follow-up duration, 29% of the VVS patients suffered at least one recurrent syncopal episode. Based on Cox regression analyses, the number of previous syncopal episodes before treatment was confirmed as a predictor of the syncopal recurrence.

VVS is common in children, and the final goal of all treatments is to terminate the unpredictable recurrence of syncope. To date, no therapeutic approaches have been documented to completely control syncopal recurrence (17). Therefore, a great number of studies have been devoted to mapping prognosis and finding predictors for syncopal recurrence (18). Variable recurrence rates from 19% to 78% were reported during a mean follow-up duration of 1.5–6 years under different situations (nonpharmacological or pharmacological interventions). In this study, the recurrence rate of 29% during a median follow-up duration of 9.0 (4.8, 19.1) months was in accordance with the results of previous studies (19–23), but our study focused on the VVS cases while on the metoprolol intervention in patients of young ages.

In the literature, many studies have suggested the number of previous syncopal episodes before treatment as a common risk factor for syncopal recurrence (4, 19–23). The findings in the present study also confirmed this, with an increase of 2.7% in the probability of recurrence for each 1-time increase in the number of previous syncopal episodes. Moreover, in the present study, we, for the first time, identified the number of previous syncopal episodes, instead of the HUTT indexes, as a predictor for syncopal recurrence in VVS children treated with metoprolol and patients with over 4 previous syncopal episodes before treatment significantly impacted the recurrence survival over time. The Kaplan–Meier curves showed that the syncopal recurrence-free survival rate over time in patients with >4 previous syncopal episodes was significantly lower than that in patients with ≤4 episodes, suggesting that the severity of the illness impacted the prognosis of the patients. The severe cases likely had a relatively high syncopal recurrence rate at follow-up. The heterogeneity of the studied populations might explain why other cutoff values were also determined (4, 21). The number of previous syncopal episodes is capable of reflecting the severity of VVS to some extent. It shows the paroxysmal trait and provides a rational clue to predict recurrence or not.

Girls in childhood and adolescence suffer from VVS twice as often as boys (24). Furthermore, females are believed to experience recurrent syncope much more often than males (21, 22). A similar sex ratio (females accounting for 69%) to that from previous reports was found in our research, but no significant difference in recurrence proportion was observed in females and males.

The QT interval is a parameter derived from an electrocardiogram, which reflects the period of ventricular repolarization. QTd implies the electrical instability of myocardia and exhibits the degree of interlead spatial variability. Kula et al. found that pediatric VVS patients with positive HUTT results had higher QTcd in the morning and late night than those with negative HUTT results and healthy controls (25). Xue et al. observed that pediatric VVS patients with positive HUTT results had higher supine QTd and QTcd values than healthy volunteers, but in VVS patients, both QTd and QTcd decreased significantly when transferring from supine to standing (26). Therefore, we analyzed QTd and QTcd in this study, but no differences were found between patients with and without recurrence, suggesting that electrical instability did not significantly impact the syncopal recurrence in VVS cases treated with beta-adrenoceptor blocker.

Inheritance in the pathophysiology of VVS should not be ignored because a relatively high ratio of positive family history was observed (27, 28). Its role is strengthened by relevant gene encoding results (29). Tanriverdi Yilmaz S et al. found a higher recurrence rate in patients with a positive family history (23). Although 17% of the patients in our study had syncope-affected families, those with syncopal recurrence did not show a higher probability of a positive family history.

Both syncope and allergic conditions are common at young ages. Du’s team found that approximately 26% of included pediatric VVS patients were affected by allergic diseases, and there were some clinical differences between VVS patients with and without allergic diseases (30). However, the underlying mechanisms underlying the relationship between allergic condition and VVS have not been fully illuminated. Our results did not show any distinction in syncopal recurrence between patients with and without allergic diseases.

Actually, there were some limitations in this study. The recall bias would impact the results for that the study was carried out retrospectively. The relatively small number of participants limits the extrapolation to other subjects. In the future, the understanding of the predictors of the long-term prognosis of children with VVS merits further large sample-sized multi-center clinical studies.

Conclusively, the present study showed that the predictor for syncopal recurrence in VVS children treated with metoprolol was the number of previous syncopal episodes. Recognition of the predictors of the prognosis of children with VVS would be beneficial to patients, relatives and physicians.

## Data Availability Statement

The original contributions presented in the study are included in the article/supplementary material, further inquiries can be directed to the corresponding authors.

## Ethics Statement

The studies involving human participants were reviewed and approved by the Ethics Committee of Peking University First Hospital. Written informed consent to participate in this study was provided by the participants’ legal guardian/next of kin.

## Author Contributions

CT had primary responsibility for the protocol development, patient enrollment, data collection, preliminary data analysis, and wrote the draft. BX analyzed the data together and revised important content. YL assisted with the study design, data collection, data analysis, and draft editing. XL designed the data analysis process and reviewed and revised the manuscript. HJ gave important advice on study design, supervised the data collection, and reviewed the manuscript for important intellectual content. JD supervised the design and execution of the study, checked the data analysis, contributed to the writing of the manuscript, and had a final approval of the manuscript submitted. All authors read and approved the final manuscript and agreed to be accountable for all aspects of the work.

## Conflict of Interest

The authors declare that the research was conducted in the absence of any commercial or financial relationships that could be construed as a potential conflict of interest.

## Publisher’s Note

All claims expressed in this article are solely those of the authors and do not necessarily represent those of their affiliated organizations, or those of the publisher, the editors and the reviewers. Any product that may be evaluated in this article, or claim that may be made by its manufacturer, is not guaranteed or endorsed by the publisher.
